# The multifaceted role of post-translational modifications in macrophage polarization: from mechanisms to therapeutic targets

**DOI:** 10.3389/fimmu.2025.1749857

**Published:** 2026-01-05

**Authors:** Rui Guo, Feng Qi

**Affiliations:** Tianjin Medical University General Hospital, Tianjin, China

**Keywords:** disease treatment, immune regulation, macrophage polarization, nanotechnology, post-translational modification

## Abstract

Macrophage polarization is central to immune homeostasis and disease pathogenesis. It is achieved through complex regulatory networks mediated by post-translational modifications (PTMs). This review synthesizes the roles of phosphorylation, ubiquitination, methylation, acetylation, and lactylation in shaping polarization outcomes through signal-responsive and metabolism-sensitive molecular networks. We integrate evidence that PTMs form interconnected circuits between signaling, epigenetic, and metabolic pathways, thereby enabling sophisticated immune interpretation. Therapeutically, we demonstrate that targeting PTM hubs rather than isolated pathways has transformative potential for reprogramming macrophages in cancer, inflammatory disorders, and tissue repair. However, applying these insights to clinical practice will require overcoming key challenges related to targeting specific pathogenic PTMs with precision, delivering them to specific cells, and validating their mechanisms *in vivo*.

## 1 Introduction

Macrophages, phagocytic immune cells derived primarily from bone marrow monocytes, are essential components of the innate immune system (1). Upon migrating into tissues, they are converted to macrophages, which are essential in achieving tissue homeostasis and triggering immune responses (2, 3). Macrophages are broadly classified into two main phenotypes: classically activated (M1) and alternatively activated (M2) macrophages (4). M1 macrophages contribute to the clearance of pathogens through the secretion of cytokines like TNF-α and IL- 6, which promote pro-inflammation. Nevertheless, their activity may also prevent cell proliferation, as well as lead to tissue damage. Conversely, M2 macrophages are implicated in the containment of the parasite and repair of tissue whereby they generate anti-inflammatory cytokines such as IL-10 to promote tissue remodelling. However, excessive M2 activation may lead to fibrosis, tumor immune evasion, and metabolic disorders (3, 5).

Macrophage differentiation into distinct functional phenotypes is guided by signals from the microenvironment. This plasticity which allows the timely adaptation to environmental changes goes beyond the simplistic M1-M2 dichotomy (6). It entails a complex cross-regulation using microenvironmental stimuli, epigenetic, metabolic re-programming, and signaling structures (7). This plasticity enables macrophages to play the role of both protective and pathogenic actions in a range of time and space. Thus the specific control of the macrophage polarization has become a central part of treatment of infections, autoimmune conditions, fibrosis and cancer along with providing novel targets in precision medicine (8, 9).

**Figure 1:**
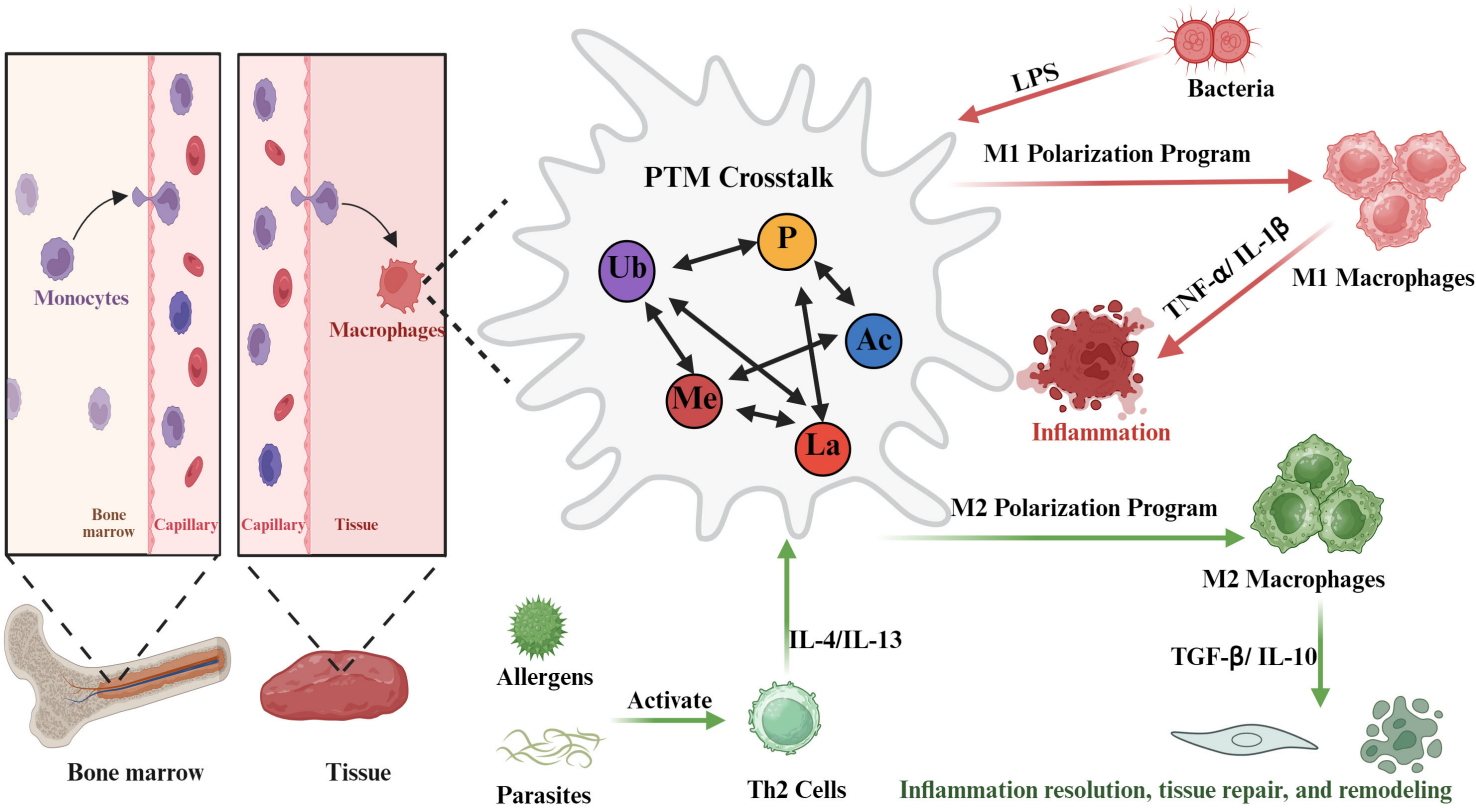
PTM crosstalk: a key regulator of macrophage plasticity. Monocytes are released from the bone marrow into the bloodstream and migrate to tissues, where they differentiate into macrophages. Macrophage polarization is influenced by different stimuli: exposure to LPS, typically derived from bacteria, promotes the M1 polarization pathway, yielding pro-inflammatory M1 macrophages. Conversely, under allergen or parasite stimulation, Th2 cells produce factors such as IL-4 or IL-13, thereby promoting the M2 polarization pathway, generating M2 macrophages associated with inflammation resolution, tissue repair, and remodeling. Cross-talk between post-translational modifications plays a key regulatory role in macrophage plasticity. (Created with Biorender.com.).

Post-translational modifications (PTMs) have become of particular importance in the research of the mechanisms of macrophage polarization in recent years (10). PTMs can either be covalent attachments of particular chemical groups (like phosphate, acetyl, ubiquin or lactate) to amino acid side chains or protein ends, or be proteolytic cleavage following synthesis by the ribosome.PTMs of proteins such as phosphorylation (11), ubiquitination (12), methylation (13), acetylation (14), and lactylation (15) can be applied singly or in combination, resulting in highly complex regulatory loops that play essential roles in regulating macrophage polarization. Indicatively, in innate immune responses, pattern recognition receptors (PRRs) undergo dynamic regulation by phosphorylation and ubiquitination which accurately modulate the action of transcription factors including NF-κB and IRFs. This regulates macrophage polarization, immunological intensity and assists in autoimmune injury prevention (16). Polarization of tumor-associated macrophages (TAMs) is strictly linked to disease progression in the tumor microenvironment. Research indicates that the PTMs have the ability to shape TAM phenotypes, which in turn affect the development, invasion and spreading of tumors (17, 18). On the same note, during inflammatory metabolism diseases, the PTMs are involved in the polarization of macrophages and influence the general immune responses (19). The usefulness of PTMs, therefore, is not limited to the physiological scenario but covers wide-ranging pathological conditions, which indicates extensive and vast influence on the biology of macrophages (**Figure 1**). Individually, studies on PTMs related regulatory mechanisms would enhance the comprehension of the process of macrophage polarization and explore the applicability of PTMs in the process and the related diseases in various physiological and pathological states. Lastly, we discuss emerging therapeutic strategies targeting PTMs for the treatment of related diseases.

## 2 Key post-translational modifications regulating macrophage polarization

### 2.1 Phosphorylation in macrophage polarization

Macrophage polarization is one of the significant characteristics of immune plasticity which is spatiotemporally regulated through the activation of different signalling pathways. The most common form of post-translational modification is phosphorylation (11). It acts as a molecular switch, playing a pivotal role in cellular processes by determining the activity, stability, protein-protein interactions, and subcellular localization of proteins. Nevertheless, over the past decade, differential transcriptomic analysis has identified thousands of genes differentially expressed between M1 and M2 macrophages (20). The fact that a large proportion of these are regulated by phosphorylation underscores the ubiquity and significance of this modification (21). Because of its fast reversible and amplifiable character, phosphorylation is highly specific in balancing the state of macrophage polarization according to varying microenvironmental signals.

A wide range of signalling pathways influence macrophage polarization,with NF-κB, JAK/STAT and PI3K/AKT pathways being the most significant. NF-κB pathway especially has a central role in the regulation of the innate immunity and inflammatory responses (22). Inflammation causes activation of Toll-like receptors (TLRs) by the presence of pathogen-associated molecular patterns (PAMPs) which cause the IKKα/β-mediated phosphorylation and ensuing degradation of IκB. This process releases the NF-κB dimer. This enables nuclear translocation and IKKα/β-mediated phosphorylation of the NF-κB p65 subunit at Ser536 and IRF3, thereby enhancing their transcriptional transactivation capacity. This culminates in the sustained activation of NF- κB and IRF3 and rapid increase in pro-inflammatory cytokines, including IL-1β, TNF-α, CCL2 and CXCL2 (23). Simultaneously, the MAPK/ERK pathway enhances the expression of inducible nitric oxide synthase (iNOS) by inducting phosphorylation on transcription factors ELK1. This promotes the synthesis of nitric oxide (NO) and reactive oxygen species (ROS) thus facilitating the change of phenotype M0 to M1. This activation occurs via the adaptor protein MyD88 which connects TLRs with IKK complexes to constitute a core signalling platform (24, 25). It is noteworthy that this synergy between the MAPK and NF-κB pathways is not merely a simple functional superposition, but rather constitutes a robust network. The complexity of the crosstalk between the MAPK and NF-κB pathways is exemplified by direct molecular interactions. For instance, the MAPK downstream kinase MSK1 directly phosphorylates the NF-κB p65 subunit. Conversely, NF-κB activation initiates a critical negative feedback loop by transcriptionally upregulating MAPK phosphatases such as MKP-1 (26). This bidirectional cross-talk creates a dynamic, interconnected network that establishes a “functional compensation” mechanism between pathways. This often renders single-target therapeutic strategies ineffective due to signal bypassing. On this basis, interventions like Nordalbergin that simultaneously inhibit multiple pathways play a crucial role in effectively achieving macrophage phenotypic reprogramming (27). Despite the compelling appeal of such multi-target drugs, they also raise new scientific questions and clinical challenges. From a scientific perspective, Nordalbergin’s broad-spectrum action makes it difficult to pinpoint which specific phosphorylation events its efficacy stems from, hindering precise identification of key therapeutic targets. From a clinical translation perspective, multi-target drugs often carry unpredictable off-target effects and may have narrow therapeutic windows. Therefore, future research strategies should focus on achieving effective macrophage phenotype conversion while mitigating the potential risks associated with multi-target drugs.

JAK/STAT signal directs macrophage polarization with high specificity by integrating signalling of extracellular cytokines. Under M1 polarization, LPS or IFN-γ stimulates JAKs associated with the receptor (JAK1, JAK2 and TYK2) resulting in the phosphorylation of STAT1 at Tyr701 and Ser727. This promotes dimerisation, nuclear translocation and DNA-binding activity that promotes the expression of inflammatory mediators like IL-1β and IL-6 (28, 29). Under M2 polarization, IL-4 and IL-13 causes JAK1/JAK3-mediated Tyr641 phosphorylation of STAT6, which is followed by second phosphorylation on Ser707. This plays a vital role in maximisation of transcriptional activity and increasing the expression of genes like Arg1, Fizz1 and CD206 (29, 30). Suppressors of cytokine signalling (SOCS) tightly and dynamically suppress this pathway. Elevated SOCS3 suppresses the phosphorylation of STAT and inflammatory production through a direct inhibition of JAK kinase activity and receptors degradation (31). Conversely, SOCS deficiency, especially SOCS3, may cause a hyperactivation of STAT which causes chronic inflammation or tumor-immune evasion. Moreover, STAT3 is long considered as one of the main regulator of macrophage polarization (32, 33). Paradoxically, under IL-10 drive, p-STAT3 promotes M2 polarization; whereas p-STAT3 driven by IL-6 family cytokines is often associated with M1 polarization (34). This paradox indicates that its ultimate biological effect is not determined solely by phosphorylation itself, but rather jointly induced by upstream triggering signals, coexisting activities of other pathways, and specific cellular microenvironments.

PI3K/AKT pathway is an important mediator of macrophage polarization,integrating signals from a variety of receptors, including cytokines and growth factors (35). Upon activation, PI3K phosphorylates membrane lipids to generate PIP3, which recruits and phosphorylates AKT at Thr308 and Ser473.Phosphorylated AKT subsequently mediates polarization responses via downstream effectors like mTOR that facilitates M2 polarization by sensitizing metabolic reprogramming to oxidative phosphorylation and regulating the activity of NF-κB and other transcriptional factors (36). Other signalling modules such as JNK pathway (37) and TGF-β/SMAD pathway (38), are added to the more complicated, multi-input regulatory network that regulates macrophage phenotype switching, and which ensures plasticity and context-sensitive reactions.

**Figure 2:**
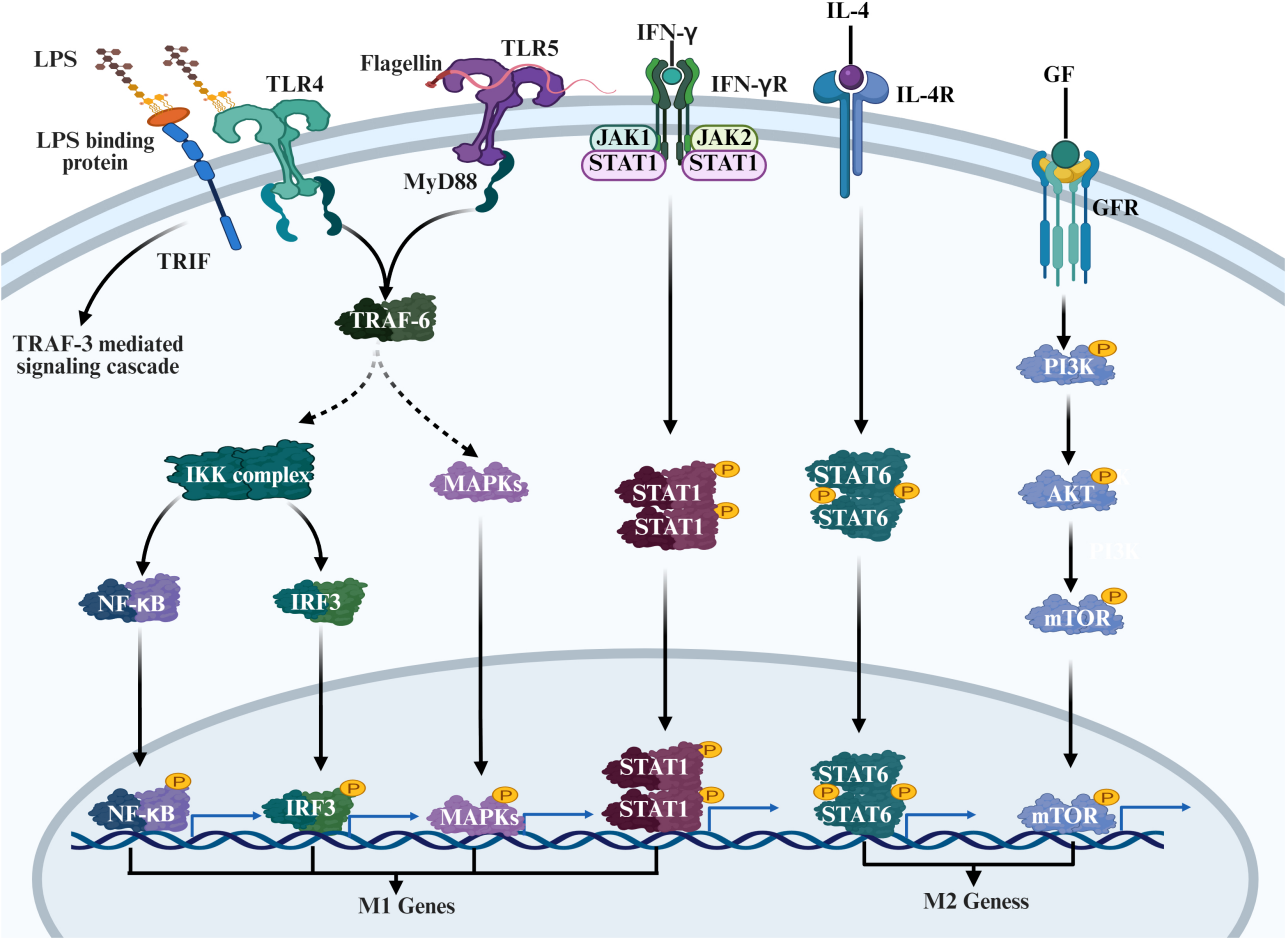
This diagram systematically illustrates the core signaling pathways governing macrophage functional polarization. M1 polarization (left) is initiated by innate immune signals: LPS binds to the TLR4 receptor, connecting to the downstream IKK complex via myeloid differentiation primary response protein 88 (MyD88). This pathway synergistically induces proinflammatory factors and M1-characteristic gene expression through the NF-κB/MAPK pathway and the TRF3 pathway, respectively. In contrast, M2 polarization (right) is dominated by adaptive immune signals. IL-4 activates the JAK1-STAT6 cascade via its receptor and engages in cross-talk with the PI3K-Akt-mTOR signaling pathway. Together, they regulate the expression profile of M2-characteristic genes, thereby mediating immune regulation and tissue repair. Additionally, IFN-γ precisely fine-tunes the polarization state via the JAK-STAT1 axis. (Created with Biorender.com.).

Therefore, in brief, macrophage polarization is regulated by several interdependent signalling pathways, where phosphorylation forms the basic regulatory element that comprises dynamic, specific and reversible regulation (**Figure 2**). The future challenge lies in utilizing more sophisticated tools to achieve precise targeting of phosphorylation functions within the body, developing strategies that can accurately intervene in specific functional modules rather than entire pathways, thereby propelling immunotherapy into a new phase.

### 2.2 Ubiquitination in macrophage polarization

Ubiquitination is a fundamental and highly reversible post-translational modification that not only fine tunes the stability and activity of key signalling molecules central to determine macrophage fate choices, but also protein-protein and macromolecular complex assembly. This specific regulation is provided through the actions of E3 ubiquitin ligases and deubiquitinating enzymes (DUBs). All of these enzymes form an important regulatory layer in the process of macrophage polarization, which makes the immune activity strong and self-restraining (12). It is noted that E3 ligases are molecular switches whereby they activate or inhibit different polarization pathways through catalyzing addition of ubiquitin moieties in either a monomeric or polymerized form with different structures. DUBs on the other hand counteract these effects by selectively cutting the ubiquitin chains to allow specific regulation of the signalling amplitudes, length and cessation. This is necessary to avoid excessive or insufficient responses to inflammation.

The E3 ubiquitin ligases have been implicated in the regulation of the balance between the pro-inflammatory M1 and anti-inflammatory M2 phenotype, through ubiquitination of central signalling nodes and transcription factors, including those in the NF-κB and STAT families. This is usually stimulus and context dependent allowing personalized immune reactions. Case in point, TRIM23 facilitates K27-linked ubiquitination, a type of chain linked to signal transduction, to promote NF-κB activation directly during viral infection,thereby facilitating a robust antiviral M1 response (39). TRAF6 is a multifunctional adaptor protein with intrinsic E3 ligase activity, that can serve as a potent inflammatory amplifier in numerous autoimmune environments. This is done through mediating K27-ubiquitination that leads to the activation of the upstream kinase TAK1. This phosphorylates and activates the IKK complex in its turn. This eventually results in translocation of NF- kB and transcription of pro-inflammatory genes including TNF-α and IL-6 (40). However, TRIM59 suppresses peripheral inflammation and M1 polarization by facilitating SUMOylation (a form of ubiquitin-like post-translation modification)to inhibit STAT1 phosphorylation. This inhibits the transcription of iNOS and the production of NO. This is one of the major feedback mechanisms that ensure that M1 hyperactivation and injuries to collateral tissue are avoided (41). On the M2 side, TRAF3 induces alternative M2 polarization which enhances ubiquitination of STAT6 at a particular residue (K450). This action causes dimerization, nuclear translocation and activity of transcription, which causes the expression of classic M2 markers (42). At the same time, a diverse array of E3 ligases including FBXW7 (43), JVT-1 (44), MAEA (45), SPOP (46), TRIM21 (47) and TRIM50 (48) inhibit M2 polarization by targeting key pro-M2 proteins, such as transcription factors, surface receptors or metabolic enzymes, for K48-linked polyubiquitination and subsequent proteasomal degradation.This has the real effect of inhibiting mechanisms like tumour immunity escape and fibrotic reactions which are dependent on M2-like macrophages. This is notably eminent in the tumour microenvironment where therapeutic strategies involving the inhibition of CDC20 or the blocking of autophagy can restore the levels of PBRM1 protein. This in turn shifts the balance towards M1 dominance and enhances responsiveness to immunotherapy. This molecular mechanism occurs due to the fact that CDC20, a part of the anaphase-promoting complex/cyclosome (APC/C), is used to recognise PBRM1 as an autophagy substrate, disaggregating it through the ubiquitination of K48 and degradation. This eliminates a check on M1 polarization and promotes an immunosuppressive M2 phenotype (49).

The tissue-specific functions of E3 ligases further increase the complexity of regulatory networks. Existing studies indicate that TRIM29 is highly expressed in mouse alveolar macrophages and negatively regulates their activation (50). However, in viral myocarditis, this molecule participates in pathogenesis by enhancing PERK-mediated endoplasmic reticulum stress responses (51). This demonstrates the tissue-dependent regulation of macrophage polarization by E3 ligases. Notably, PERK signaling itself serves as a key metabolic switch driving M2 macrophage polarization (52). Based on these findings, we propose a forward-looking scientific hypothesis: Could TRIM29 ubiquitinate PERK, thereby coupling endoplasmic reticulum stress signaling with macrophage polarization states (particularly toward the M2 phenotype) under specific pathological conditions such as infection or tissue injury? Although this model remains unproven by direct experimental evidence, given TRIM29 regulatory role in diseases affecting multiple tissues including the respiratory system (50), gut (53), and heart (51), exploring its potential holds significant inspire value.

There is a direct antagonistic function of deubiquitinating enzymes that counteracts the activity of E3 ligases by selective deubiquitination. This introduces another key of dynamic control, fine-tuning of signalling outputs, signal resolution with the removal of stimulus and cellular homeostasis. An example is that USP1 and USP18 promote M2 polarization and tumour progression by deubiquitinating and stabilizing attractive receptors, including CXCR4 and CSF1R respectively in tumour-associated macrophage. This increases chemotactic responses and survival signalling and eventually strengthens the pro-tumoural functions of the macrophage population (54, 55). Conversely, USP25 impairs M2 polarization by interacting with STAT6 to reduce K48-linked ubiquitination of STAT6. This suppresses the STAT6/PPAR-γ signalling and restricts expression of the M2-related genes, where USP25 is a possible endogenous inhibitor of alternative activation (56). It is worth noting that not all ubiquitination processes cause degradation through the proteasomal system, and the functional consequence is heavily dependent on the type of ubiquitin chain linkage. One such example is TRAF6 which regulates IL-4-induced M2 polarization through non-degradative action. TRAF6 is recruited to the STAT6 complex upon IL-4 stimulation and catalyses K63-linked polyubiquitination of STAT6. This particular change does not cause degradation of STAT6, but it helps to shield it against proteasomal recognition to increase its protein half-life and enhance its accumulation in the nucleus. It also promotes transcription of classic M2 markers like Arg-1, Fizz1 and Ym1 by the STAT6-Arg1 and STAT6-Mrc1 axis (57). This demonstrates the flexibility, and even antagonism, of the same E3 ligase in regulating the polarization of macrophages, depending on the cellular context, the nature of the stimulus, and the ubiquitin linkage type that it catalyses.

In summary, ubiquitination acts as a versatile and powerful molecular switch that directs the polarisation of macrophages towards either the pro-inflammatory M1 or the anti-inflammatory M2 phenotypes. This is done by conjugating important signalling proteins with certain ubiquitin chains and as such control their fate, function and interactions. The complex, context-dependent and dynamically regulated interaction between the activating actions of E3 ligases and the reversing activity of deubiquitinases (DUBs) results in an effective but versatile control system. This provides abundant repertoire of possible therapeutic targets of immunomodulatory therapy across a broad spectrum of diseases, such as cancer, chronic inflammation, autoimmune diseases and fibrotic diseases.

### 2.3 Methylation in macrophage polarization

Methylation modifications, which are important post-translational modifications of proteins and nucleic acids, have a far-reaching impact on polarization-relevant gene expression, modulation of key signalling pathways, and on individual protein functions (58). This regulatory system can be categorised into two distinct but interconnected classifications: histone methylation (13), which primarily establishes permissive or repressive chromatin states to control the transcriptional accessibility of polarization genes; and non-histone methylation, which directly regulates the functional activity, stability, and interaction networks of signalling proteins involved in phenotypic switching (59). Methylation mark can be interpreted as conveying entirely opposite polarity instructions across different genetic contexts, leading to the potential for therapeutic strategies targeting methylation enzymes to encounter the dilemma of epigenetic antagonism.

This dilemma is particularly pronounced in the regulation of histone methylation. G9a, recognised as the primary histone lysine methyltransferase in mammalian cells, is chiefly responsible for depositing H3K9 methylation marks. These marks have been well-documented to be related to transcriptionally repressive chromatin states and gene silencing. However, its polarizing effect is entirely dependent on the targeted gene sites.It has been shown that G9a stimulates H3K9 methylation in the promoter region of forkhead box protein P1 (FOXP1) which causes compaction of chromatin and transcriptional inhibition that in turn causes M1 macrophage polarization by removing this differentiation brake (60). Moreover, G9a-mediated H3K9 methylation exerts a significant influence on M2 macrophage polarization by selectively suppressing IRF4 gene transcription, and thereby strategically inhibits the expression of this essential M2-transcription factor and provides an epigenetic block to alternative activation (61). This indicates that the same methyltransferase plays antagonistic roles in the polarization network by regulating different genes. Another specialised histone methyltransferase which catalyzes H3K9 methylation is domain-containing protein 1 (SETDB1) that has been reported to directly bind and activate the promoter of monocarboxylate transporter 1 (MCT1). This transcriptional activation has been demonstrated to stabilise the levels of HIF-1α protein thereby triggering a typical M2 polarization program and related angiogenesis (62). The opposing applications of G9a and SETDB1 to the same histone mark reveal a fundamental characteristic of methylation regulation: its function is not determined by the mark itself, but by the specific gene carrying that mark. In glioma stem cells,the enrichment of histone H3K4 in one active chromatin mark,the CD47 promoter region, is catalysed by AMPK,thus transcriptionally upregulating this powerful “don’t eat me” signal. After recognizing the CD47 by the macrophages surface signal-regulating protein alpha (SIRPα), an intracellular phosphorylation cascade involving Src homology 2 domain-containing protein tyrosine phosphatase-1 (SHP-1) is initiated, significantly impairing the phagocytic capacity of macrophages and suppressing their anti-tumour M1 functions. Moreover, the CD47-SIRPα-SHP-1 signaling pathway has been shown to have a concomitant inhibitory effect on the activation of NF-κB/STAT1 pathway,thereby reinforcing the maintenance of macrophages in M2-like immunosuppressive state (63). In addition to the mentioned particular examples, the role of methylation at numerous additional histone sites, such as H3K27 (64), H3K36 (65) and H4K20 (66), collectively contribute to the outcome of macrophage polarization.

Non-histone methylation extends this regulatory logic from the chromosomal level to the signal transduction level. Methyltransferase 21A (METTL21A) is a significant lysine methyltransferase that targets non-histone substrates. Recently, it has been found to cause tumour progression in hepatocellular carcinoma (HCC) through methylating and stabilising BCL2-associated antiapoptotic gene 3 (BAG3). This stabilization is inextricably linked with the establishment of an M2-polarised microenvironment by tumour-associated macrophages because BAG3 enhances the survival-signalling and cytokine networks that are conducive to alternative activation. Notably, the METTL21A inhibition has been shown to revitalize BAG3 degradation, trigger M1 macrophage reprogramming, and improve the efficacy of sorafenib, which offers the novel combined Methylation and molecular chaperone intervention approach to the treatment of hepatocellular carcinoma (67). Moreover, lysine-specific demethylase 1 (LSD1) has also been found to be a major regulatory factor due to its function in reducing NF-κB p65 methylation through a process of lysine demethylation. This decreased DNA methylation event increases p65 transcriptional activity, thus expressing JNK/NF-κB signaling, responding to elevated percentages of M1 macrophages, and leading to intense inflammatory reactions (68). This positioning of LSD1 as a critical switch in inflammation-mediated polarization control is therefore evident.

It should be mentioned that histone and non-histone methylation are not independent pathways. Instead, they are seen as highly interactive regulatory layer that together combine to define the pathway of macrophage polarization by keeping the epigenetics and post-translational coordination. The core mechanism by which methylation regulates macrophage polarization lies in its site-specific epigenetic coding. Future therapeutic strategies should not be limited to simply inhibiting a specific methyltransferase or demethylase, the true breakthrough lies in developing tools that can precisely target specific pathogenic gene sites.

### 2.4 Acetylation in macrophage polarization

Acetylation is a pivotal post-translational modification that dynamically regulates macrophage polarization through precise control of gene expression programs and signalling pathway activities. The advanced “write-erase-read” paradigm is the functional basis of histone acetylation, and it allows rapid reconfiguration of the state of transcriptional factor accessibility and chromatin structure (69). This regulatory system is exhaustive and has emerged as a future therapeutic target to control macrophage phenotype and macrophage functionality in a diversity of disease conditions,offering many points of intervention pharmacological access.

Acetyltransferase family regulates the process of “writing” (i.e. the addition of acetyl groups to specific lysine residues on both histone and non-histone proteins). N1-acetyl-L-histidine, which is released into the extracellular microenvironment in the hepatocellular carcinoma, is selectively endocytosed by tumour-associated macrophages and directly triggers the acetyltransferase EP300 by allosteric modulation. The activation of this process instigates widespread H3K27 acetylation at promoter regions, consequently leading to the augmentation of a family of immunosuppressive genes, such as Arg1, IL-10, and PD-L1. This mechanism has been shown to enhance effective M2 polarization, and inhibit CD8+ T cell cytotoxic function and impair anti-PD-1 therapeutic effect (70). It should be mentioned that all these effects can be fully reversed when a particular EP300 is inhibited. Through the relevant literature, it is evident that the inhibitory effect of small-Molecule inhibitors on the activity of acetyltransferases has been described as an effective measure in the process of restoring immunotherapy following the occurrence of both nasopharyngeal (71) and gastric (72) cancers. This repair is done through reprogramming the balance of the macrophage polarization. In the context of various inflammatory conditions, such as LPS stimulation, histone acetyltransferases EP300 have been found to mediate the entire TLR4/MAL/MyD88/NF-κB signalling pathway to achieve effective M1 polarization (73). This is a mechanistically supported route that has been widely confirmed in a variety of disease models such as psoriasis (74), rheumatoid arthritis (75), autoimmune arthritis (76) and myocardial inflammatory responses (77). The distinct polarization trends generated by EP300 primarily depend on which signaling pathways recruit it to specific genomic regions. This mechanism also explains why EP300 inhibitors effectively restore immunotherapy efficacy in nasopharyngeal carcinoma and gastric cancer models. They likely selectively block abnormal acetylation processes within the tumor microenvironment without broadly inhibiting EP300 essential functions in physiological immune responses.

The “erasing” process involves the specific elimination of acetyl groups with some of the numerous groups of deacetylases, which facilitates the reversibility of the signal with great precision and dynamic regulation. Histone deacetylases are classified in four different classes depending on subcellular localization and other functional features. HDAC1, 2, 3, 8 are nuclear-based enzymes of class I (78). The current work examines how HDAC2 supports the process of M2 polarization by deacetylation of the transcription factor SP1 in the K703 site specifically. This action assists in stable interactions of SP1 to M2 gene promoters, such as those of Arg1, Mrc1 and IL-10, and also elicits the activation of STAT3 phosphorylation (79). The metabolites of the tumor microbiome containing butyrates inhibit this process well since it suppresses the enzyme activity of HDAC2. Though it is possible that in the case of complex microbial dysbiosis in progressive tumours, this can eventually promote M2 polarization and tumour recurrence (80). The nuclear SPHK2/S1P signalling has been tested in models of LPS-induced acute lung injury and it can inhibit HDAC1/2-mediated p53 deacetylation, leading to sustained p53 hyperacetylation, bursts of ROS in the mitochondria, and activation of the NLRP3 inflammasome. The procedure causes pro-inflammatory amplification loop (81). Class II HDACs have unique characteristic of being capable of shuttling into the nucleocytoplasm including two different subtypes Class IIa (HDAC4, 5, 7, 9) and Class IIb (HDAC6, 10) (78). The present study examines how HDAC4 contributes to the metastasis of breast cancer. It shows that HDAC4 promotes the deacetylation of NEDD9 promoter region, which in turn promotes NF-κB signalling and M2 polarization (82). In colorectal cancer models, HDAC6 activates the TAK1-ADAM17 axis, amplifying soluble IL-6 receptor release to drive M2 polarization and confer chemoresistance (83). Moreover, it is shown that HDAC10 directly deacetylates STAT3, thus facilitating M2 polarization and Th2-inflammatory response in the asthma model of allergies (84). Class III deacetylases (SIRT1–7) (85) include the mitochondrially localised SIRT3,that has been reported to regulate TFAM acetylation and release, resulting in CCL4/CXCR5 elevation and M1 polarization which has been discovered to be effective in suppressing tumour growth in a variety of cancer types (86). It has been identified that endothelial-specific SIRT3 deficiency enhances mitochondrial ROS–NF-κB signalling, which promotes CCL2/IL-6 release and M1-driven adipose tissue inflammation and fibrosis in metabolic syndrome. It is important to mention that this pathological process is reversible due to the restoration or activation of the SIRT3 (87). The only Class IV HDAC11 was shown to control IL-10 level by modulating promoter expression thus inducing systemic immune tolerance in transplantation models (88). Different classes of HDACs may play mutually counterbalancing roles in the polarization process by regulating distinct substrates. Consequently, the use of broad-spectrum HDAC inhibitors may lead to counterproductive therapeutic effects, underscoring the advantages of targeted inhibitors.

The “reading” phase involves the specific recognition of acetylation marks by specialized protein domains, such as bromodomains, which selectively recruit or exclude downstream transcriptional complexes to regulate gene expression outcomes (89). The basic idea is also applied in the innovative technology of proteolysis-targeting chimera (PROTAC) that provides an additional new degradation-based system that is used to regulate these readers. As an example, the competent ARV-825 molecule has been found to bind with cereblon E3 ubiquitin ligases, which leads to the selective degradation of BRD4. The process has been demonstrated to induce great antitumour effects in various ways. Moreover, it is known that ARV-825 has some benefits compared to the traditional bromodomain inhibitors regarding the dosing frequency used, the profiles of side effects, and the development of resistance (90).

**Figure 3:**
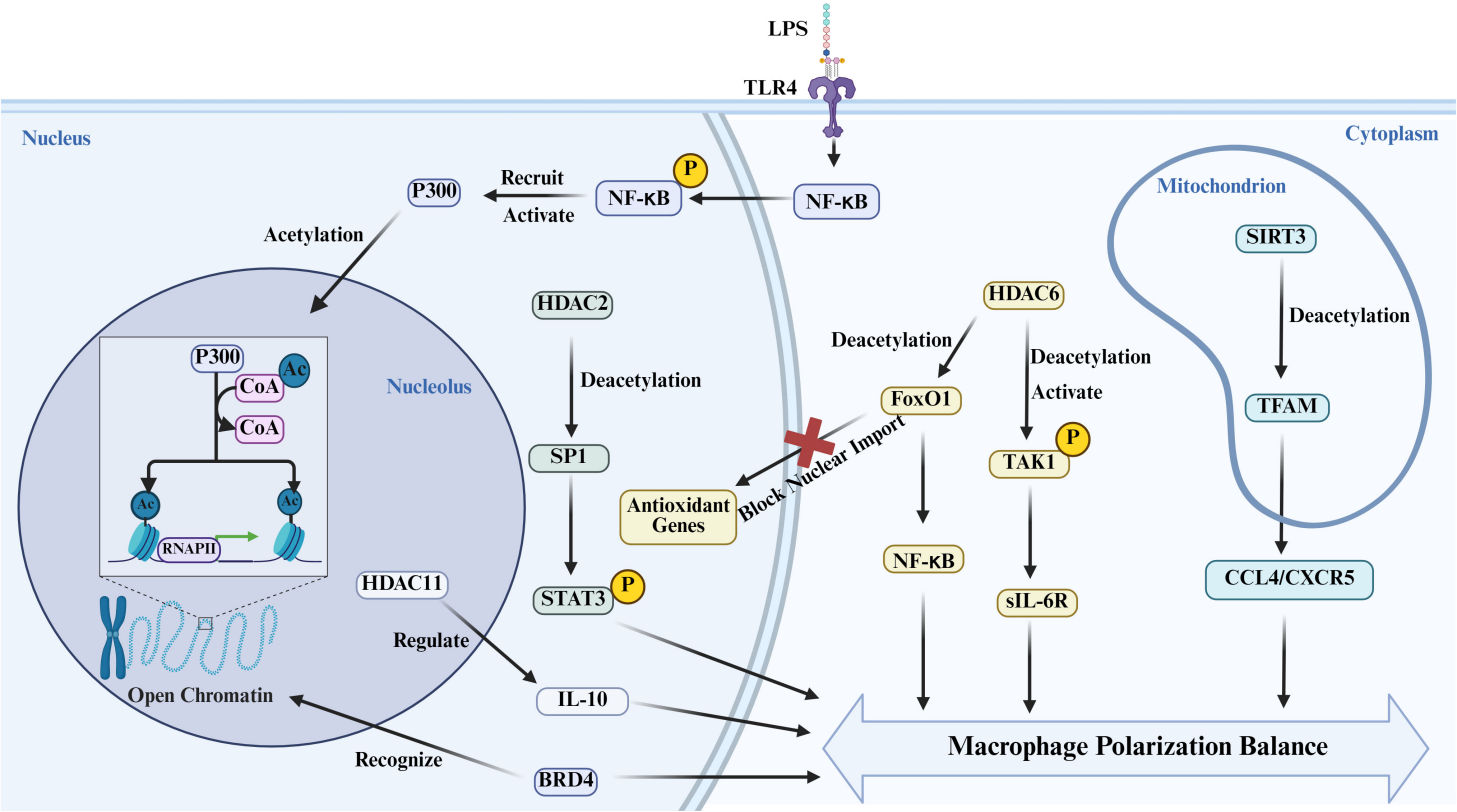
The dynamic process of epigenetic regulation involves “writing, erasing, and reading.” Upon stimulation by inflammatory signals such as LPS, NF-κB is activated and translocates to the nucleus, where it recruits and activates the histone acetyltransferase P300 to perform “writing” modifications on histones. Histone acetylation promotes gene transcription by loosening chromatin structure. This modification can be “read” by the bromodomain-containing protein BRD4, thereby initiating inflammatory reprogramming in macrophages. Simultaneously, histone deacetylases (HDACs) act as “erasers,” precisely regulating the direction of macrophage polarization by removing acetylation modifications at specific sites. (Created with Biorender.com.).

To sum up, the “write-erase-read” cycle is a dynamic regulatory axis controlling decisions on macrophage polarization on several levels, providing a thriving array of therapeutical targets in the treatment of inflammatory diseases and cancer (**Figure 3**). Future mechanistic work on such regulatory pathways and their crosstalk could lead to the development of novel methods of specific immune modulation and such personalised therapy of a variety of human diseases.

### 2.5 Lactylation in macrophage polarization

Lactic acid that was traditionally perceived as a byproduct of anaerobic metabolism has been identified as a key regulatory molecule in inflammatory and tumour microenvironment. It also acts as an energy substrate as well as signalling mediator (91, 92). The ability of this process to covalently modify lysine residues on proteins,a process termed “lactylation” has revealed a previously unknown mechanism of connection between cellular metabolism and epigenetics through a direct biochemical relationship. This modification was proposed in the seminal 2019 Nature report on histone lysine lactylation, as an obligatory point of connection between metabolic reprogramming, chromatin-scale epigenetic adjustment, and functional immune polarization, providing new insights into the way the microenvironmental signal combines metabolic data into gene expression programs (15).

**Figure 4:**
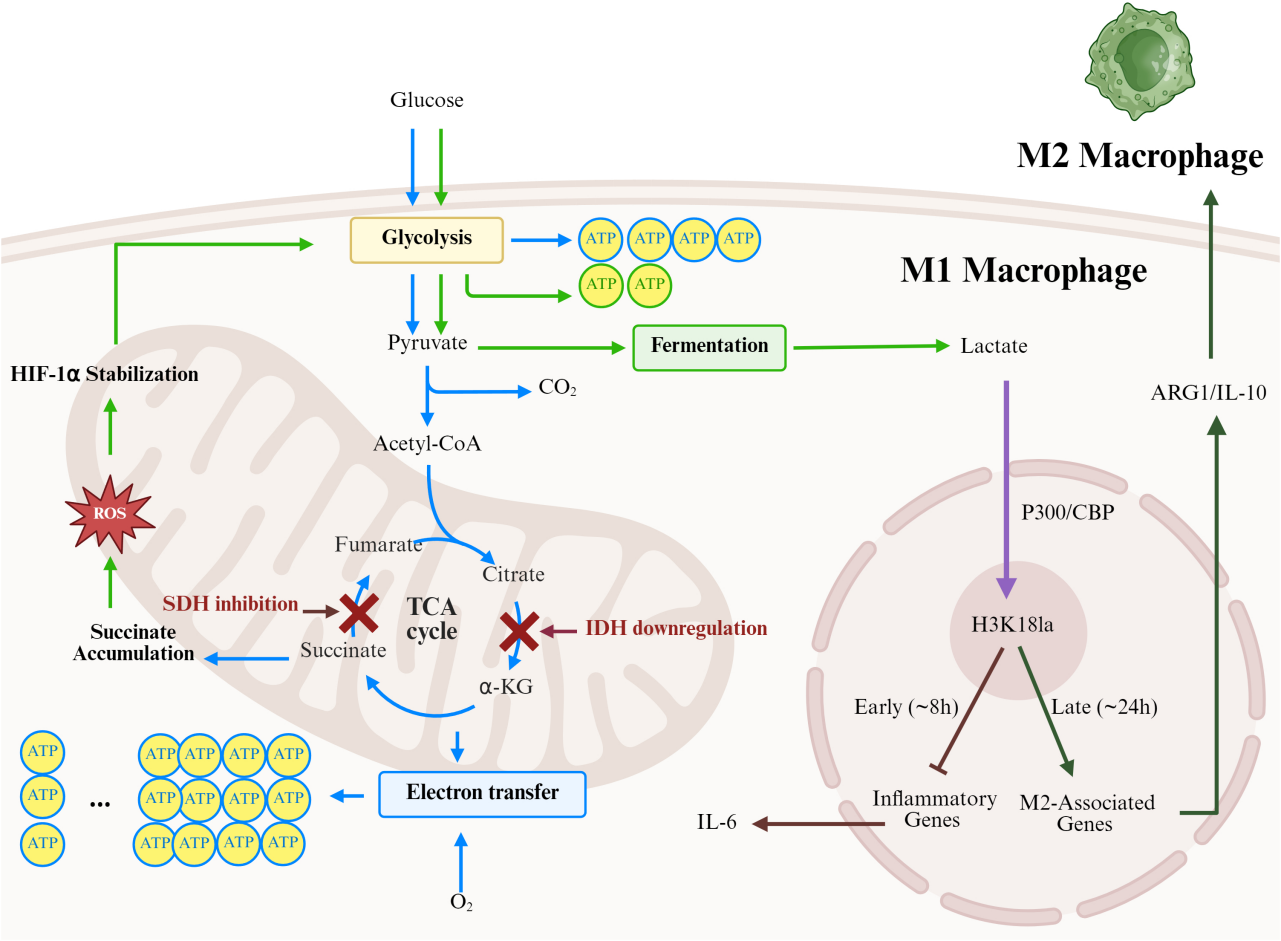
“Lactic acid timer” modulates the inflammatory and reparative processes of macrophages. The polarization of M1 macrophages is accompanied by significant metabolic reprogramming. The tricarboxylic acid cycle is interrupted at citrate and succinate, which leads to the accumulation of these metabolites and intensified glycolysis via the ROS-HIF-1α axis. This results in substantial lactate production. The generated lactate serves as a substrate for the lactoylation modification of H3K18, which is catalysed by P300 and dynamically regulates gene transcription programmes. This lactoylation modification suppresses inflammatory responses in the early stages while initiating repair processes in the later stages. This dual-phase regulatory mechanism, known as the “lactic acid timer”, precisely controls the transition of macrophages from a pro-inflammatory to a pro-repair state. (Created with Biorender.com.).

The separation of metabolic characteristics between various macrophage subsets indicates specialised lactate dynamics which are correlated with the functional state of the various macrophages subsets. The proinflammatory M1 macrophages have been noted to depend on aerobic glycolysis strongly,with high glucose consumption rates that leads to lactate build up despite sufficient oxygen concentrations. This is referred to as the Warburg effect. Conversely, anti-inflammatory M2 macrophages show an affinity to oxidative phosphorylation and fatty acid oxidative metabolic processes, thus keeping a lower level of intracellular lactate through enhanced mitochondrial metabolism (93). It has been shown that there was a direct effect of lactate on the role of macrophages via the mechanism of lactylation of histone, which is concentration dependent. The rapid activation of glycolytes in the M1 macrophages can lead to the production and accumulation of lactate when stimulated by LPS. This, in turn, increases the level of global histone lactylation by non-enzymatic chemical reactions. In conditions of sustained high lactate levels, prevalent in chronic inflammation and tumours, this modification has been shown to activate M2-associated tissue repair genes, such as Arg1 and IL-10, through the creation of a permissive chromatin environment. This implies that lactate levels might be used as a composite metabolic signaling change to promote a progression of early inflammation to resultant tissue repair. This idea has been called “lactic acid timer”, which relates the metabolic state to phenotypic advancement (15) (**Figure 4**). In-depth analysis reveals that the functional output of lactylation exhibits high signal context dependency. Across different disease models and cell types, identical lactylation modifications can drive entirely distinct transcriptional programs. This makes direct intervention in lactate metabolism or lactylation modifications likely to produce unpredictable off-target effects.

The exact translocation of lactate across cell membranes occurs with the assistance of specialized monocarboxylate transporters (MCTs), with different isoforms directing lactate flux in specific directions to maintain metabolic homeostasis (94, 95). MCT1 has been shown to be able to mediate the uptake of the extracellular lactate to activate the STAT6 signalling pathway in the presence of also preventing the activity of NF-κB to induce M2 polarization (96). This process is demonstrated to support the repair mechanisms in the pancreas after an episode of acute pancreatitis through the regulation of macrophage phenotyping (97). In contrast, MCT4 mainly mediates the excretion of lactate through high glycolytic cells. Its selective inhibition in macrophages results in significant intracellular lactate accumulation, subsequent H3K18 lactylation enrichment at specific genomic loci, and ultimately enhanced M2 polarization through a process of transcriptional reprogramming (98). These specialised transport systems make MCTs key spatial regulators of the cellular lactylation environment by regulating the supply of substrate to this modification.

The functional role of lactylation, which occurs especially at the H3K18 site, is notably to be applied with consistency across a variety of pathological situations. The expression of hexokinase 2 (HK2) in metabolic dysfunction-linked steatohepatitis (MASLD) represents a connection between elevation in glycolytic flux and lactate production, which triggers IL-10 expression together with NF-κB signalling via H3K18la-promoting chromatin opening. Nuclear-translocated NF-κB also transcriptionally regulates HK2 expression whereby this forms a positive feedback loop of disease progression making it a metabolism-inflammation positive feedback loop (99). Similarly, in the case of silicon dioxide nanoparticle induced pulmonary fibrosis, the p300-mediated H3K18 lactylation has a direct effect on the persistent M1 polarization as mediated by the programming of specific genes (100). This change is also tactically exploited by tumor microenvironments: in gastric cancer (101), this modification using lactate to induce H3K18 lactylation, induces VCAM1 expression, subsequently activating AKT-mTOR-CXCL1 signaling to recruit and polarize M2 macrophages, whereas in ovarian cancer (102), this modification is used to activate M2 polarization by CCL18 upregulation,facilitating immune evasion and metastasis. This indicates that the functional output of lactate production is not solely determined by lactate concentration but is also influenced by its subcellular distribution and metabolic pathways. The intense competition for lactate between cancer cells and immune cells further complicates this process.

Recent studies have revealed that microbial metabolites can directly remodel the host’s epigenetic landscape (103). Enriched anaerobic bacteria in colorectal cancer tissues produce lactate in large quantities through the diol-pyruvate pathway, which leads to a dramatic rise in tumour lactate levels beyond the normal glycolytic production. It has been found that tumor-associated macrophages actively absorb this lactate of bacterial origin through certain MCTs. This in turn causes the activation of histone acetyltransferases p300/CBP to catalyse H3K18 and H3K9 lactylation. Simultaneously, NF-κB-induced inflammatory signalling is repressed thus trapping these macrophages in a stable M2 phenotype and creating an immunosuppressive microenvironment that is highly suppressive of tumor growth and progression (104, 105). This provides a novel mechanism for understanding tumor immune evasion and suggests that regulating the gut microbiota may offer a new strategy for indirectly intervening in lactylation.

Taken together, these varied studies make lactylation, and more specifically, H3K18 lactylation, as a crucial molecular signal that translates metabolic signals into precise epigenetic instructions on the choice of macrophage polarization. The conserved mechanism has been seen to work in a variety of conditions, such as acute and chronic inflammation, fibrotic diseases, and several types of cancers. This poses the idea that H3K18 lactylation can potentially have a high level of potential both as a dynamic biomarker and a therapeutic target. It is particularly promising in its ability to modulate pathological immune responses in diverse disease contexts through metabolic-epigenetic interplay.

### 2.6 Interactions among multiple post-translational modifications in macrophage polarization

The major post-translational modifications, such as phosphorylation, ubiquitination, methylation, acetylation, and lactylation are not cascade-like during the process of macrophage polarization. Rather, they communicate via common signalling nodes and form integrated regulatory routes to afford denoted spatiotemporal regulation over signalling intensity, duration, and termination (16, 106). This, further, dictates the dynamism of the balance between the pro-inflammatory and the anti-inflammatory phenotype. To gain a deeper understanding of the operational logic of this integrated network, it is necessary to systematically compare the major PTMs driving macrophage polarization across dimensions such as chemical properties, kinetics, and functional focus (**Table 1**). These interactions primarily follow three core logics: sequential coordination and feedback regulation, antagonism and equilibrium, and metabolic-epigenetic checkpoint control. The following sections will elaborate on how these patterns are achieved through metabolic coupling modifications such as the TAK1-IKK-NF-κB cascade and lactylation.

**Table 1:** Cross-regulation of PTMs at key nodes of macrophage polarization.

Interaction patterns	Core node	Key PTM events and mechanisms	Functional output and polarization effects
Sequential Coordination and Positive Feedback	NF-κB p65	Phosphorylation (by IKK) initiates activation; Acetylation (by p300/CBP) enhances transcription and inhibits its degradation.	Potently drives and sustains M1 polarization; Forms a positive feedback loop.
	TAK1	K63-linked ubiquitination (by TRAF6) triggers activation; Phosphorylation-acetylation cascade provides feedback stabilization.	Integrates upstream signals and amplifies inflammatory output.
Antagonism and Dynamic Equilibrium	STAT1	Activating phosphorylation (by JAK) drives M1 program; Inhibitory ubiquitination (e.g., by TRIM59) serves as a “molecular brake”.	Precisely controls M1 response intensity, preventing excessive inflammation.
	STAT6	Phosphorylation (by JAK) initiates M2 program; K63 ubiquitination (by TRAF6) enhances activity; Lactate metabolically activates its signaling axis.	Ensures robust M2 polarization via multiple inputs.
Metabolic-Epigenetic Coupling	Histone H3K18	Lactylation is directly driven by microenvironmental lactate; competes with acetylation etc., for the same lysine residue.	Acts as a “metabolic timer”, directly translating metabolic status into instructions for polarization switching.

One example of this complex crosstalk is the TAK1-IKK signalling hub that is an essential integration node of several modification types. Upon stimulation by TNF-α or IL-1β, TAK1 is rapidly recruited to receptor complexes and undergoes K63-linked polyubiquitination at specific lysine residues. E3 ligase TRAF6 in cooperation with UBC13 ubiquitin-conjugating enzymes can usually catalyse this process. The particular ubiquitination allows TAK1 to become activated, and with this change in conformation, it undergoes autophosphorylation resulting in the appearance of docking sites with which to bind its downstream substrates. The activation of TAK1 has been demonstrated to promote the assembly of the IKK complex that in turn triggers the phosphorylation of the NF-κB p65 subunit on serine 536. This facilitates the transcriptional action of NF-κB (107). This phosphorylation event establishes the necessary molecular context for subsequent acetylation by recruiting histone acetyltransferases such as p300/CBP, which acetylate p65 at multiple lysine residues (108). It should also be noted that the p65 acetylation has been found to increase the DNA binding affinity and also stabilise the TAK1 by inhibiting its K48-linked ubiquitin-dependent degradation, thereby forming a positive feedback loop. This coordinated modification cascade has been shown to extend pro-inflammatory gene expression and strengthen M1 polarization by supporting the continued activity of NF-κB (22). On the other hand, at the stage of resolution, deacetylases such as SIRT1, take away these acetyl groups on a time-dependent basis, facilitating TAK1 degradation through renewed ubiquitin-proteasome system recognition and shifting macrophages toward an anti-inflammatory repair phenotype. This demonstrates how sequential modifications create a molecular timer for inflammatory responses (109, 110).

The other advanced type is on the metabolite-based modifications that directly link cellular metabolism to epigenetic programming. The IL-4-induced M2 polarization causes a high glycolytic flux, with the rapid elevation of intracellular lactate levels that, in turn, causes the non-enzymatic reaction between histone H3K18 lactylation. This in turn stimulates M2-specific genes, including Arg1and Mrc 1, through the opening of chromatin architecture at their promoters. As polarization goes on and metabolic programs evolve, TCA cycle restoration causes succinyl-CoA accumulation by increasing the metabolism of glutamine and anaplerotic flux. This metabolic shift has been found to facilitate the H3K122 succinylation through histone succinyltransferases. This dynamic mechanism of sequential modification provides a natural braking system that restrains the excessive polarization of M2 and makes sure that the tissue repair responses are within a healthy calibration (111). The active interactions between activating lactylation and repressive succinylation represents a highly complex metabolic-epigenetic checkpoint that optimally adjusts phenotype outcomes based on the availability of different metabolites and mitochondrial activity, thereby preventing overshooting of either inflammatory or reparative responses.

Taken together, these interacting processes form a complex “superimposed-antagonistic” regulatory network, in which modifications either cooperate synergistically to amplify signals or compete sterically and functionally to shape macrophage plasticity with remarkable precision. This combined approach clarifies the constraints of single-target therapy and highlights the significant possibility of multi-target therapeutic interventions that can simultaneously tune multiple modification pathways to maximally control macrophage activity in an infection, cancer, metabolic inflammation, and tissue repair situation and provide better response at minimum compensatory mechanisms.

## 3 PTM-based therapeutic strategies for macrophage polarization

Macrophages are extremely functional and phenotypically adapted which is why they have vital and multifaceted roles in the development of diseases. The current research postulates that the delicate equilibrium between pro-inflammatory M1 and anti-inflammatory M2 polarization states exerts a significant influence on pathological outcomes of a variety of pathologies, among them, but not limited to, chronic inflammation, tissue repair mechanisms and tumour immunology (5, 6). Over the last twenty years, studies efforts have been largely focused on understanding the role of cytokines and transcription factors as the major determinant of macrophage polarization. Many of these pathways however, have been limited by pleiotropic toxicity, short molecular half-lives and emerging resistance mechanisms (112). Novel and specific immune modulation with greater specificity has become possible by the rapidly growing repertoire of knowledge about post-translational modifications (PTMs),including phosphorylation, ubiquitination, methylation, acetylation, and the newly emerged method of lactylation (113). By strategically targeting specific PTM regulatory nodes within macrophage signalling networks, it is increasingly possible to reprogramme polarization trajectories with spatial and temporal precision, offering a promising and innovative strategy for targeted therapeutic interventions in inflammatory diseases, fibrotic disorders, and cancer immunotherapy with potentially reduced off-target effects.

### 3.1 Drug therapy targeting post-translational modifications

It has already been demonstrated that pharmacological modulation of post-translational modifications (PTMs) is actually a potential and future approach to fine-tuning of macrophage polarization and providing fresh prospects of immunomodulatory therapy in immune-mediated diseases. Several targeted agents have the capacity to demonstrate this potential through molecular mechanisms that are distinct yet effective (**Table 2**). The oral JAK1/2 inhibitor Baricitinib which has been clinically approved for the treatment of rheumatoid arthritis, has been demonstrated to induce M2 polarization significantly by directly inhibiting JAK1/STAT3 phosphorylation (114). Anti-inflammatory effects of baricitinib have been proven with certainty in the case of experimental myocarditis models (115). A pharmaceutical compound that is more commonly used in the treatment of central nervous system lymphoma is Ibrutinib, which is a Bruton tyrosine kinase (BTK) inhibitor (116). In sepsis models, ibrutinib has been shown to effectively drive M2 polarization through the robust inhibition of BTK activation and subsequent NF-κB phosphorylation. At the same time, it has also been noted to regenerate the platelet count by using an unprecedented mechanism, thereby providing an excellent dual-target therapy agent in the treatment of sepsis-related thrombocytopenia (117). Multi-target tyrosine kinase inhibitor Crizotinib has been demonstrated to affect the metabolism of lactate through inhibiting the CD147-MCT1 molecular interactions. It has been shown to lead to a large decrease in the level of H3K18 lactylation as well as the expression of CXCL13. This co-efficiency step qualitatively inhibits the M2 polarization alongside boosting the cytotoxic ability of CD8+ T cell, and this eventually limits the growth of melanoma and increases greatly the anti-PD-1 immunotherapy effectiveness (118). Cetuximab, which targets the epidermal growth factor receptor (EGFR), has been approved and is routinely used in the treatment of various malignant tumors, such as head and neck cancer (119). Similarly the investigational MST1R inhibitor WM-S1–030 has been shown to greatly improve the antitumor effects of cetuximab by reprogramming M2 macrophages to M1 via phosphorylation inhibition (120), though it remains in active preclinical development stages. As can be seen, there are also several other promising investigational agents that have shown a significant potential of augmenting anti-PD-1 immune response effectively.These agents include Eliprodil (121), the potent AhR antagonist CH223191 (122), and innovative ARPC1B-targeting siRNAs (123). It is however important to note that such candidates are still on the initial stages of exploration research.

**Table 2:** Drugs targeting PTMs.

Category	Intervention	Key molecular mechanism	Polarization outcome	Functional outcome	Disease model
Targeted Agents	Baricitinib	JAK inhibitor; Suppress STAT3 phosphorylation	M2 ↑	Anti-inflammatory	Rheumatoid Arthritis; Myocarditis
	Ibrutinib	BTK inhibitor; Suppress NF-κB phosphorylation	M2 ↑	Anti-inflammatory	Sepsis
	Crizotinib	Tyrosine Kinas Inhibitor;Reduce H3K18 lactylation	M2 ↓	Anti-tumor; Enhance anti-PD-1 efficacy	Melanoma
	WM-S1-030	MST1R inhibitor; Inhibit phosphorylation	M1 ↑,M2 ↓	Anti-tumor; Enhance cetuximab effects	Head and Neck Cancer; Colorectal Cancer

At the same time, Traditional Chinese Medicine (TCM) offers a complex “multi-component, multi-target” holistic treatment of the macrophage reprogramming through integrated epigenetic, metabolic, and PTM-mediated regulatory mechanisms (124) (**Table 3**). Berberine, a well-characterised isoquinoline alkaloid derived from Coptis chinensis, has been shown to differentially regulate macrophage polarization through a dual mechanism (125). First, it stimulates the autophagy pathways that are dependent on the p85/AKT/mTOR and at the same time, it inhibits MAPK/NF-κB signaling cascades. This regulation of core metabolic and stress-signaling pathways directly impacts the activity of associated kinases and metabolic sensors like AMPK, which can subsequently influence the activity of histone acetyltransferases (HATs) and deacetylases (HDACs), thereby altering the epigenetic landscape. This results in the coordinated downregulation of M1 markers (IL-6, TNF-α) while promoting M2-associated mediators (IL-10, TGF-β). This, consequently, works in the effect of relieving gastroesophageal reflux disease pathology (126, 127). Paradoxically,the ample antitumor effects of berberine have an astonishing dose-dependent effect as they markedly suppress the phosphorylation and release of the STAT3 to increase the prevalence of M1 macrophages in tumor microflora (125). Tripterylin is an organic molecule extracted as Tripterygium wilfordiiroot bark and has been shown to have a great therapeutic potential in model of autoimmune nephropathy and rheumatoid arthritis. Its mechanism involves the modulation of polarization through a complex dual-axis pathway, involving the suppression of the NF-κB/NLRP3-glycolysis axis, while concurrently activating AMPK-oxidative phosphorylation pathways (128). Ginkgolide, which has a long-standing tradition of application in the management of cardiovascular and neurological diseases, has been demonstrated to be particularly effective in preventing p65 phosphorylation and, thus, the excessive polarization of M1 (129). Oroxylin A has been demonstrated to demonstrate remarkable precision by specifically targeting the critical p62-TRAF6 interface to effectively block TRAF6 oligomerization and K63 ubiquitination, thereby inhibiting the NF-κB/NLRP3 inflammatory axis in psoriasis models (130).

**Table 3:** TCM targeting PTMs.

Category	Intervention	Key molecular mechanism	Polarization outcome	Functional outcome	Disease model
TCM Agents	Berberine	Activate p85/AKT/mTOR dependent autophagy; Inhibit MAPK/NF-κB phosphorylation; Suppress STAT3 phosphorylation	M1 ↓(low doses) M2 ↓(high doses)	Anti-inflammatory at low doses; Anti-tumor at high doses	Gastroesophageal Reflux Disease
	Tripterylin	Suppress NF-κB/NLRP3- glycolysis axis; Activate AMPK-oxidative phosphorylation	M1 ↓, M2 ↑	Anti-inflammatory	Autoimmune; Nephropathy; Rheumatoid arthritis
	Ginkgolide	Inhibit p65 phosphorylation	M1 ↓	Anti-inflammatory	Cardiovascular and Neurological Diseases
	Oroxylin A	Block TRAF6 oligomerization and K63-linked ubiquitination; Inhibit NF-κB/NLRP3 axis	M1 ↓	Anti-inflammatory	Psoriasis
	Polysaccharide-Iron Nanozyme Platform	Scavenge ROS; Reduce HIF-1α stability; Modulate H3K9me3/ H3K4me3	M2 ↑	Anti-inflammatory and tissue repair	Inflammatory bowel disease with anemia

With advancing TCM-nanotechnology, new innovative therapeutic formulations are being produced. One of the most promising self-assembled polysaccharide-iron nanoproducts has been found (131). It combines peony or goji berry polysaccharides with Fe^3+^ to form uniform 200 nm spherical nanocomposites. This channel demonstrates strong SOD/catalase cascade activity that fulfills the rapid scavenging of ROS, decreases the protein stability of HIF-1α, and suppresses the glycolytic flux. This broad redox regulation is able to regain KDM4B demethylase activity, dramatically reducing H3K9me3 repressive marks on TNF-α and IL-6 promoters, but also significantly increasing H3K4me3 activating marks on IL-10 and Arg-1 gene loci, which would effectively promote M2 polarization. This novel paradigm combines inherent immunomodulatory activity of herbal polysaccharides and the unparalleled catalytic capacity of advanced nanozymes to offer a novel strong therapeutic platform in the management of inflammatory bowel disease with its associated problems of anemia.

Other proven effective natural compounds, such as baicalin (132), alpinia galanga (133), panax notoginseng saponins (134), and shikonin (135), all inhibit M1 polarization in a variety of disease models such as acute lung injury and myocardial ischemia-reperfusion. Meanwhile, betulinic acid (136), liensinine (137) and glycyrrhetinic acid (138) can be used to successfully reprogram tumor-associated macrophages (TAMs) phenotype from protumor M2 to antitumor M1, and combinations of these agents with anti-PD-1 immunotherapy can potently inhibit tumor growth in a wide range of cancer models.

In summary, both modern targeted agents and traditional TCM compounds have been shown to successfully achieve precise macrophage reprogramming through sophisticated multi-modal PTM regulation, convincingly demonstrating substantial therapeutic potential for treating inflammatory diseases, cancer, and tissue repair conditions. However, translating this into clinical practice still faces core challenges: first, the issue of target specificity,how to precisely intervene in disease-related PTMs without disrupting physiological functions. Second, the resulting risk of off-target effects. Crucially, the third challenge concerns *in vivo* delivery specificity: macrophages exhibit high heterogeneity. Precisely delivering regulatory molecules to specific subpopulations at the lesion site (such as tumor-associated macrophages) while avoiding non-selective uptake by other cells represents a critical bottleneck determining both efficacy and safety. This is precisely why nanotechnology and metabolic regulation strategies hold immense promise, offering revolutionary tools and approaches to overcome these delivery and specificity limitations.

### 3.2 Nanotechnology

Research has increasingly demonstrated that targeted delivery of post-translational modification (PTM) modulators enables precise spatiotemporal regulation of macrophage polarization, thereby offering a novel therapeutic approach for immune-related disorders. The utilisation of artificial nanoparticles (NPs) and extracellular vesicles (EVs) has emerged as a highly effective delivery platform, with each exhibiting distinct yet complementary biological mechanisms for modulating macrophage phenotypes through controlled release and specific targeting strategies.

The outcome of polarization induced by nanoparticles is found to be remarkably dependent on their finely tuned physicochemical characteristics, including material composition, hydrodynamic size, surface charge and properties, and strategic functional modifications (139, 140). For instance,a study with Selenium-chlorogenic acid nanoparticles demonstrated the concomitant delivery of Se^4+^ and chlorogenic acid, consequently neutralizing various reactive oxygen species (ROS), and inhibiting both MAPK/JNK and PI3K-AKT signalling pathways through a coordinated action mechanism.This causes the inhibition of the pro-inflammatory M1 polarization, while concurrently promoting the anti-inflammatory M2 polarization in a balanced way (141). In the complex tumour microenvironment, sophisticated multifunctional nanodrugs (NCG) have been shown to successfully remodel M1 macrophages and actively recruit CD8+ T cells by enhancing JAK1-STAT1 signalling while concurrently blocking phosphorylation of STAT6, STAT3, and AKT through multi-pathway intervention, ultimately leading to significant inhibition of tumour metastasis and progression (142). Nanoparticles are distinctly beneficial in production scalability, high drug loading capacity, and structural programmability for customized applications. Nevertheless, considerable challenges remain in ensuring long-term immunological safety profiles and effectively overcoming biological barriers for optimal tissue-specific delivery.

Contrastingly, with a very sharp contrast, extracellular vesicles,naturally occurring lipid bilayer nanoparticles of endogenous nature,have intrinsic advantages in high biocompatibility and high biological barrier penetration capability. It has been discovered that endogenous nanoparticles, including EVs, are in a position to allow essential intercellular communication of exchanging biomolecules. These EVs are enriched with source-cell-specific proteins, regulatory microRNAs and functional lipids. EVs exhibit significantly superior safety profiles and facilitate more straightforward standardisation of production, stable storage conditions, and streamlined clinical management protocols when compared to their parent cells (143). The distinguishing feature of EVs produced by mesenchymal stem cells and alternative activated M2 macrophages is the regulatory RNAs (e.g. miRNAs-124, -126 (144), -21-5p (145) and let-7a-5p (146))that they carry,which effectively suppress the NF-κB/MAPK pathway while simultaneously activating PI3K-Akt signalling through coordinated molecular actions. These actions suppress the pro-inflammatory M1 phenotype and promote M2 anti-inflammatory polarization in a physiologically relevant manner. Furthermore, the influence of tumor-derived EVs on TAM polarization dynamics is also of significance: gastric cancer EVs containing let-7g-5p have been shown to actively drive M2 polarization via STAT3 phosphorylation-mediated signaling (147), while colorectal cancer EVs carrying miR-122 have been demonstrated to substantially contribute to chemotherapy resistance through adaptive microenvironment remodeling (148). Conversely, ingeniously engineered bacterial outer membrane vesicle-based nanoplatforms (OMV@NP) actively induce M1 polarization through the TLR4/MyD88/NF-κB pathway activation, and when strategically combined with antigen and adjuvant release systems, effectively reverse tumor-induced immunosuppression by reprogramming the myeloid compartment (149).

**Figure 5:**
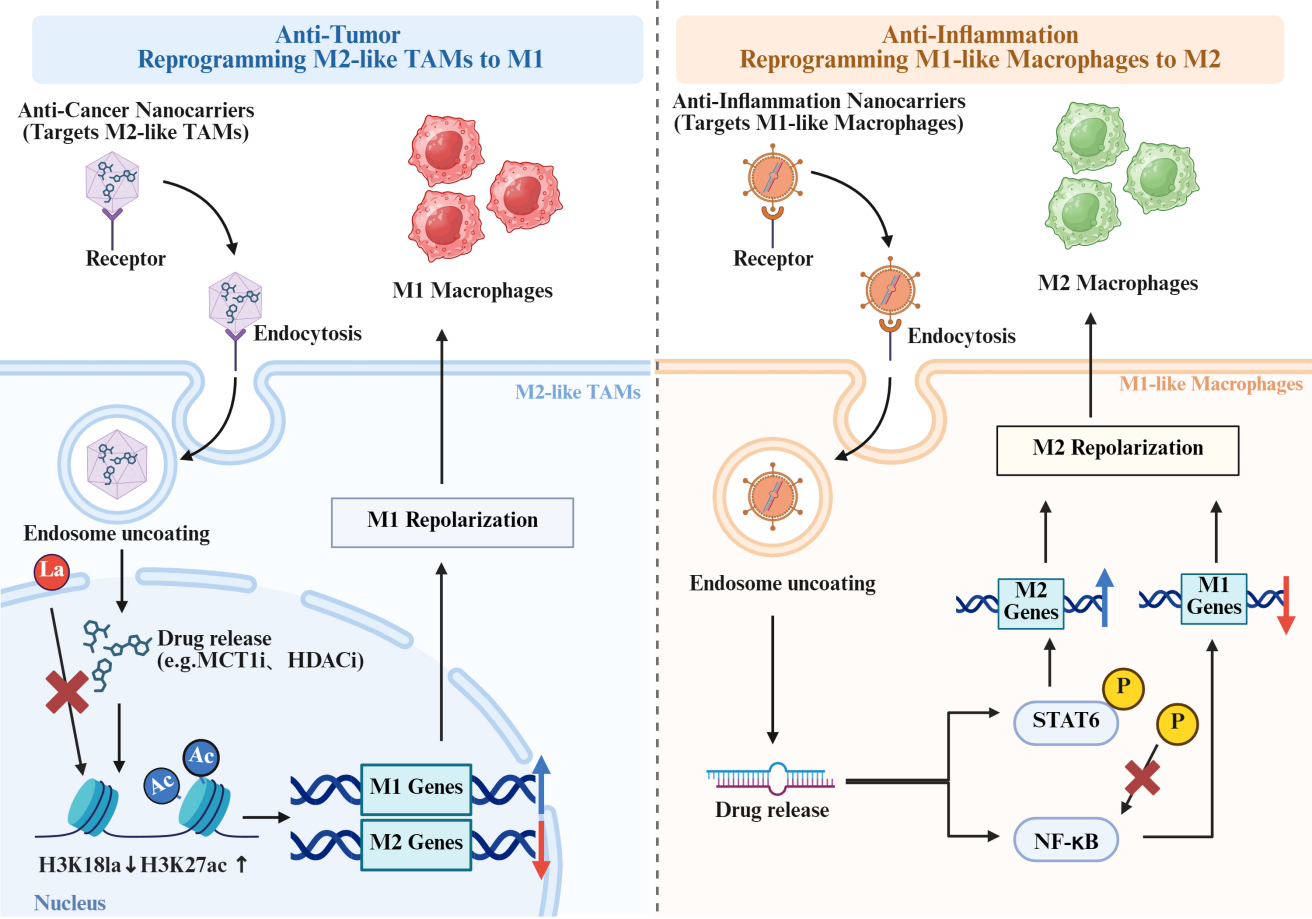
Functionalized nanocarriers drive precise immune regulation by targeting specific cell subpopulations and modulating key targets to induce their phenotypic conversion. (Created with Biorender.com.).

In summary, it is evident that advanced nanotechnology platforms, encompassing both synthetically engineered nanoparticles and naturally derived extracellular vesicles, exert a substantial influence on the polarization of macrophages (**Figure 5**). This influence is characterised by the efficient delivery of specific signalling molecules, including microRNAs (miRNAs), long non-coding RNAs (lncRNAs), and functional proteins, to target cells. These sophisticated delivery systems provide powerful and versatile tools for regulating immune responses with precision, modulating inflammatory states in a controlled manner, and effectively intervening in disease progression pathways. Collectively, these findings highlight their broad potential in next-generation immunotherapy and precision medicine applications across diverse pathological conditions.

### 3.3 Metabolic regulation

Metabolic reprogramming has been identified as a fundamental and dynamic mechanism that governs the process of macrophage polarization. It functions as a molecular switch that directs the fate of immune cells by means of precise regulation of energy metabolism pathways. During classical M1 polarization induced by inflammatory stimuli, the tricarboxylic acid (TCA) cycle undergoes characteristic disruption at two critical metabolic nodes, marked by citrate accumulation and subsequent succinate buildup. These events lead to substantial mitochondrial reactive oxygen species (ROS) production through reverse electron transport. This metabolic configuration has been demonstrated to stabilise hypoxia-inducible factor-1α (HIF-1α) protein levels and actively drive expression of pro-inflammatory genes such as GLUT1, iNOS, and IL-1β, thereby establishing a self-amplifying inflammatory metabolic program. In stark contrast, alternative M2 polarization relies predominantly on oxidative phosphorylation and fatty acid oxidation pathways, thereby maintaining TCA cycle integrity and preserving mitochondrial membrane potential to efficiently support the synthesis of tissue repair mediators such as arginine and ornithine through coordinated metabolic flux (150, 151).

The targeting of these critical metabolic nodes offers significant potential for the reprogramming of macrophage functional states through strategic metabolic intervention. The elimination of excessive ROS (152, 153), the reduction of glucose transporters GLUT1 and hexokinase HK2, and the inhibition of glycolysis, can significantly reduce intracellular lactate levels and subsequent H3K18 lactylation modifications (154). This, in turn, can disrupt NLRP3 inflammasome assembly and diminish IL-1β and IL-6 pro-inflammatory cytokine release. Concurrently, the activation of the AMPK-PGC1α axis, in conjunction with the strategic inhibition of PI3K-AKT-mTORC1 signalling, has been shown to enhance oxidative phosphorylation capacity and mitochondrial biogenesis, thereby promoting comprehensive anti-inflammatory metabolic reprogramming (155, 156). In the complex tumour microenvironment, innovative mitochondrial-targeted conjugates such as rhein-dichloroacetic acid have been shown to simultaneously block the PDK-PDH axis and mitochondrial complex I activity, thereby depleting cellular ATP stores and inducing controlled ROS storms (157). This multi-faceted metabolic disruption not only cuts off the energy supply in cancer cells, but also effectively triggers the repolarisation of macrophages from pro-tumour M2 to anti-tumour M1 phenotypes, thereby enhancing overall anti-tumour immunity through metabolic immunotherapy.

The application of advanced nanotechnology and biophysical approaches has led to the development of increasingly precise metabolic intervention strategies. Electrostatic self-assembly of positively charged magnetite nanoparticles (pFe_3_O_4_) on MXene nanosheets forms a stable co-dispersed system that can be fabricated into functional biomimetic bone scaffolds (PFM). These intelligent scaffolds have been demonstrated to effectively upregulate Arg2 expression, enhance mitochondrial respiratory function, and accelerate oxidative phosphorylation rates, thereby driving M2-to-M1 conversion in bone regeneration contexts (158). Photobiomodulation (PBM), a non-invasive low-energy light irradiation technique, has been shown to directly modulate bone marrow-derived macrophage metabolism by reducing NF-κB p65 expression and phosphorylation activation, resulting in a significant decrease in M1 proportion and an increase in M2 macrophage numbers. This innovative approach has been shown to suppress ROS production, TNF-α secretion, iNOS expression, and IL-1β release, thereby effectively alleviating inflammation and tissue oedema. This has been demonstrated in a variety of inflammatory disease models with consistent efficacy (159).

In summary, metabolic reprogramming is a promising and versatile therapeutic axis for the control of macrophage polarization, with broad clinical implications. It is proposed that by strategically integrating pharmacological inhibitors, advanced nanomaterial systems, and innovative biophysical strategies, researchers can effectively transition macrophages from pro-inflammatory “war metabolism” to anti-inflammatory “rebuilding metabolism”. This offers novel and powerful avenues for immune remodelling in cancer immunotherapy, chronic inflammatory diseases, and regenerative medicine applications.

## 4 Conclusion

This review systematically synthesises the current understanding of the multidimensional regulatory roles played by diverse post-translational modifications (PTMs) in directing macrophage polarization. It comprehensively details how key modifications – including phosphorylation, ubiquitination, methylation, acetylation, and the recently characterised lactylation – collectively orchestrate the dynamic and plastic transition between pro-inflammatory M1 and anti-inflammatory M2 phenotypes through sophisticated molecular networks. Extensive documentation has been provided demonstrating that these PTMs do not operate in isolation, but rather form interconnected regulatory circuits that enable precise spatiotemporal control over signalling amplitude, duration, and specificity. This, in turn, determines the functional fate of macrophages in various pathological contexts. Emerging experimental and clinical evidence demonstrates that multiple intervention strategies can effectively modulate these PTM networks with remarkable precision. These strategies include the use of small-molecule inhibitors, biologically active natural compounds derived from traditional Chinese medicine, advanced nanomaterial-based delivery systems, and innovative metabolism-targeting approaches. These strategies have already shown promising therapeutic outcomes across diverse disease models, including infection, chronic inflammation, tissue fibrosis, and multiple cancer types through successful macrophage reprogramming.

Despite advances in both mechanisms and therapeutic interventions, critical challenges persist in this field. First, most current studies rely on *in vitro* cellular models that cannot fully replicate the complex and dynamic microenvironments found *in vivo*. This technical limitation hinders precise elucidation of the dynamic regulation of PTMs. Second, bulk sequencing techniques obscure cellular heterogeneity, and most conclusions stem from studies of bone marrow-derived or peritoneal macrophages. Whether tissue-resident macrophages possess unique PTM regulatory mechanisms, and how these distinct mechanisms determine their specialized functions, remains largely unknown. This leads to a significant underestimation of macrophage heterogeneity and tissue specificity in PTM regulation. Furthermore, the complex interactions among PTMs pose challenges when using broad-spectrum modulators, suggesting a future focus on developing therapeutic interventions that precisely target pathogenic PTMs, a paramount challenge in medicinal chemistry.

In the future, the systematic integration of cutting-edge technologies from the fields of chemical biology, nanomedicine and artificial intelligence is expected to dramatically accelerate the translation of PTM-targeted macrophage reprogramming strategies from fundamental research discoveries to clinical bedside applications. This is substantially supported by rapid advances in single-cell multi-omics profiling, AI-driven drug development platforms and spatially temporally controlled delivery technologies. Single-cell multi-omics will enable unprecedented resolution in mapping the dynamics of post-translational modifications (PTMs) during the process of cell polarization. In addition, artificial intelligence (AI) algorithms will be able to predict the most suitable intervention nodes within complex PTM networks. This will allow the design of novel modulators with enhanced specificity. This strategic interdisciplinary convergence positions PTM-based immunomodulation as a truly next-generation strategy in precision immunotherapy. The potential exists to develop personalised therapeutic regimens that account for individual patient variations in PTM enzyme expression and activity. This would ultimately enable more effective and durable treatments for immune-related disorders with minimised off-target effects. This evolving paradigm signifies a fundamental shift from conventional cytokine-centric approaches towards mastering the epigenetic and post-translational codes that govern immune cell identity and function in health and disease.
